# Improving the Solubility, Stability, and Bioavailability of Albendazole through Synthetic Salts

**DOI:** 10.3390/molecules29153571

**Published:** 2024-07-29

**Authors:** Haiying Yan, Xueping Zhong, Yao Liu

**Affiliations:** Medical College, Qinghai University, Xining 810001, China; liuweiqi0706@163.com (X.Z.); 13158395346@163.com (Y.L.)

**Keywords:** albendazole, salt, solid-state properties, solubility, stability, oral bioavailability

## Abstract

Albendazole (ABZ) is a highly effective yet poorly water-soluble antiparasitic drug known to form salts (ABZ-FMA, ABZ-DTA, and ABZ-HCl) with fumaric acid (FMA), D-tartaric acid (DTA), and hydrochloric acid (HCl). This research utilized a range of analytical techniques, including Fourier transform infrared spectroscopy (FT-IR), nuclear magnetic resonance hydrogen spectroscopy (^1^H NMR), powder X-ray diffraction (PXRD), dynamic vapor sorption (DVS), thermogravimetric analysis (TGA), differential scanning calorimetry (DSC), and scanning electron microscopy (SEM), to validate and characterize the solid-state properties of these drug salts. This study also assessed the solubility and intrinsic dissolution rate (IDR) of these salts under different pH conditions compared to the active pharmaceutical ingredient (API) and conducted stability studies. Moreover, the in vivo pharmacokinetic performance of ABZ salt was evaluated. The results of this study reveal that the new solid form of ABZ is primarily associated with amino acid esters and benzimidazole groups, forming intermolecular interactions. All three ABZ salts significantly improved the solubility and dissolution rate of ABZ, with ABZ-HCl demonstrating the optimal performance. Importantly, the drug salt exhibited robust physical stability when exposed to adverse conditions, including strong light irradiation (4500 ± 500 lux), high humidity (92.5 ± 5% relative humidity), elevated temperatures (50 ± 2 °C), and accelerated test conditions (40 °C/75 ± 5% relative humidity). Lastly, the in vivo pharmacokinetic analysis demonstrated that ABZ salt led to a substantial increase in AUC_(0–24)_ and C_max_ compared to ABZ. This elevation in solubility in aqueous solvents signifies that ABZ salt exhibits characteristics that can enhance oral bioavailability and pharmacokinetics. These findings provide potential solutions for the development of more effective and innovative drug formulations.

## 1. Introduction

Albendazole (ABZ) is an antiparasitic drug within the benzimidazole family, which is widely used in clinical practice for the treatment of several parasitic infections, including echinococcus [1,2,3]. ABZ was first approved as an anthelmintic drug for human use in 1982 [4]. It is not only a basic drug recognized by the World Health Organization (WHO) for the treatment of systemic parasitic infections but also the preferred drug endorsed by the WHO for clinical treatment of echinococcosis. ABZ is highly effective with limited toxicity, and it may have anti-tumor activity against human pancreatic cancer cells [5], gastric cancer cells [6,7], and colorectal cancer cells [8]. Therefore, ABZ has been recognized as a potential cancer treatment drug.

ABZ is a Class II drug based on the biopharmaceutical classification system, with low solubility (ABZ has a solubility of 0.0228 mg/mL in water at 25 °C) and high permeability (log P 2.54) [9,10]. The low solubility and poor gastrointestinal absorption of ABZ reduce its applicability and clinical efficacy, with an oral bioavailability in humans of less than 5% [11]. Therefore, enhancing the solubility of ABZ is essential. Some pharmaceutical approaches have been employed to try to improve the water solubility of ABZ, such as solid dispersions [12,13], nanocrystals [14,15], liposomes [16], cyclodextrins [17,18], inclusion complexes [19], and self-emulsifying drug delivery systems [20]. Nonetheless, due to low drug efficiency, an excessive dosage of surfactants, complex preparation processes, and unidentified stability, further application or industrial production of drug formulations remains limited.

In the pharmaceutical industry, using appropriate counter ions in the formation of salts is an optimal solution to increase the solubility of compounds [21]. Additionally, salt formation is a simple and easy way to scale up the process. This technique is an effective way to obtain higher solubility alongside an enhanced dissolution rate. Approximately half of the active pharmaceutical ingredients (APIs) currently approved by the US Food and Drug Administration (FDA) are salts [22]. The use of ABZ salts has gained great interest, and several ABZ salts have been created. For example, Paulekuhn et al. [23] described methods for the preparation of ABZ hydrochloride, methanesulfonate, sulfate, and toluenesulfonate, and introduced characterization studies of these solid forms. For ionizable molecules such as anions, cations, and zwitterions, salt formation is the most straightforward and cost-effective strategy for enhancing drug solubility. However, when converting raw materials into salts, consideration of temperature, humidity, and drug excipients is required to tune the physical and chemical properties of crystals, which may be positive or negative [24]. Therefore, understanding the solid-state properties of drug salts is critical for evaluating the efficacy and safety of pharmaceutically important raw materials.

**Figure 1:**
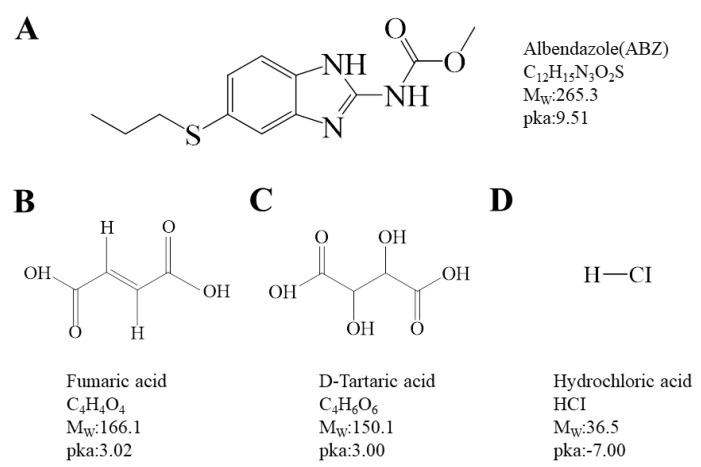
Chemical structures of albendazole (**A**), fumaric acid (**B**), D-tartaric acid (**C**), and hydrochloric acid (**D**).

Based on the ΔpKa rule [25,26], when the difference between two pKa values ((conjugate acid of a base)—(acid)) is over three, a salt can generally be formed. This study identified fumaric acid (FMA, pKa = 3.02), D-tartaric acid (DTA, pKa = 3.00), and hydrochloric acid (HCl, pKa = −7.00) from the acids of known salt-forming drugs, and prepared salts alongside ABZ (pKa 9.51) (Figure 1) to investigate changes in solubility and bioavailability. The ABZ salt was generated through solvent evaporation, with ABZ-FMA being the first identified. The formation of the salt and the molecular interaction mechanisms between ABZ and different acids were examined using diverse methods such as Fourier transform infrared spectroscopy (FT-IR), nuclear magnetic resonance hydrogen spectroscopy (^1^H NMR), powder X-ray diffraction (PXRD), dynamic vapor sorption (DVS), thermogravimetric analysis (TGA), differential scanning calorimetry (DSC), and scanning electron microscopy (SEM). The dissolution behavior of API and drug salts in aqueous solutions at various pH levels was examined through equilibrium solubility experiments, and the intrinsic dissolution rate (IDR) was assessed to characterize the improvement in the drug salt dissolution rate compared to the API. Concurrently, stability experiments were conducted under diverse conditions. Moreover, the in vivo pharmacokinetics of the salt were explored to identify the improvement in its bioavailability following oral administration.

## 2. Results and Discussion

### 2.1. Characterization

**Figure 2:**
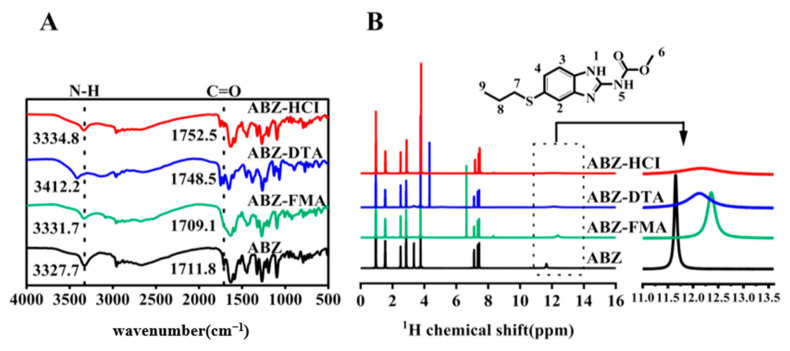
FT-IR spectra (**A**) and ^1^H NMR spectra (**B**) of ABZ, ABZ-FMA, ABZ-DTA, and ABZ-HCl.

To investigate if certain characteristic vibrational states were impacted after salt formation and the molecular mechanism of drug–acid interaction (shifts, disappearances, or changes in infrared characteristic peaks represent intermolecular interactions), we collected FT-IR spectra of ABZ, ABZ-FMA, ABZ-DTA, and ABZ-HCl. As illustrated in Figure 2A, the characteristic absorption peaks of ABZ appeared at 3327.5 cm^−1^ and 1711.7 cm^−1^, related to the vibrations of N-H and C=O, consistent with the description in a prior report [27]. Compared to ABZ, the N-H peaks of ABZ-FMA, ABZ-DTA, and ABZ-HCl in the prepared salt exhibited a red shift, reaching 3331.7 cm^−1^, 3412.2 cm^−1^, and 3338.4 cm^−1^, respectively, suggesting the presence of novel hydrogen bonds within these solids. Additionally, the C=O peak of ABZ-DTA and ABZ-HCl in the spectrum also exhibited a red shift to 1748.5 cm^−1^ and 1752.5 cm^−1^, respectively, while the C=O peak of ABZ-FMA shifted to 1709.1 cm^−1^. The characteristic absorption peaks of ABZ shifted to varying degrees following salt formation, further confirming the changes in the hydrogen-bond interaction in the salt of ABZ. This may be because of the protonation of the benzimidazole group in ABZ, alongside the shift of amide and carbonyl peak positions, providing evidence for the formation of three ABZ salts [28].

To comprehend the structure and conformational effects of functional groups in salt formation, the interaction between ABZ and various acidic protons was examined through ^1^H-NMR. Figure 2B illustrates the ^1^H NMR spectra of ABZ and salt. In addition, the amide protons of H^1^ and H^5^ in the ABZ structure are potential interaction sites. As outlined in the figure, ABZ exhibited a sharp amide peak at 11.66 ppm, and the amide peaks of ABZ-FMA, ABZ-DTA, and ABZ-HCl all shifted to 12.36 ppm, 12.12 ppm, and 12.19 ppm, respectively, with shifts of 0.69 ppm, 0.46 ppm, and 0.53 ppm relative to ABZ. The findings of ^1^H-NMR correspond to those of FT-IR, confirming the salt formation. In the new solid form of ABZ, each component predominantly interacts with amino acid esters and benzimidazole groups to generate intermolecular interactions.

**Figure 3:**
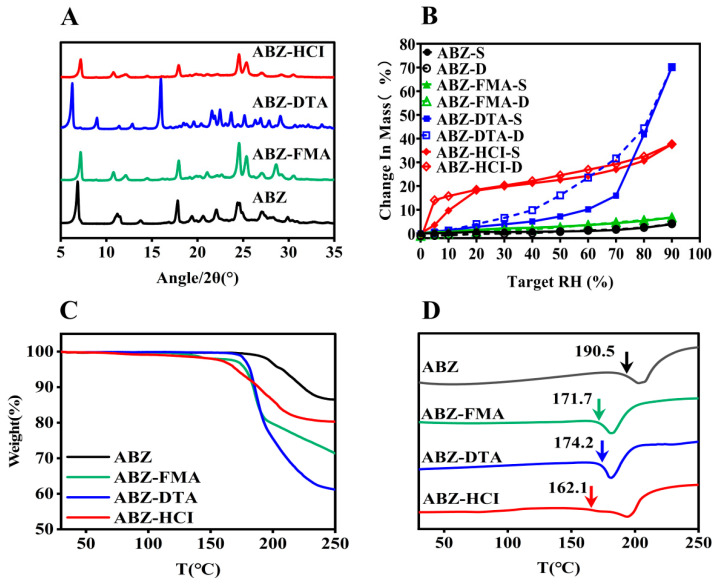
PXRD pattern illustrated by (**A**), DVS (**B**), TGA (**C**), and DSC (**D**) curves of ABZ, ABZ-FMA, ABZ-DTA, and ABZ-HCl (the temperature indicated by the arrow in (**D**) is the initial melting point, *T_m,onset_*).

PXRD is the most commonly employed technology for the investigation of crystal formation [29,30]. The PXRD patterns of ABZ and the salts (ABZ-FMA, ABZ-DTA, and ABZ-HCl) were assessed, as shown in Figure 3A. ABZ exhibited characteristic crystalline peaks at 2θ values of 6.83°, 11.76°, 17.81°, and 29.91°. ABZ-FMA exhibited characteristic crystalline peaks at 2θ values of 7.14°, 10.78°, 17.96°, and 30.52°. ABZ-DTA exhibited characteristic peaks at 6.27°, 8.98°, and 16.96°, and ABZ-HCl had characteristic peaks at 7.21°, 10.73°, and 17.91°. The differences in the PXRD patterns of the ABZ salt from the original materials indicated new crystalline phase formation. 

To assess the water adsorption characteristics of ABZ salts, DVS was employed to study the moisture adsorption isotherms of ABZ, ABZ-FMA, ABZ-DTA, and ABZ-HCl over the range of 0–90% RH. As illustrated in Figure 3B, all solid samples continuously adsorbed moisture with increasing humidity. When the RH reached 90%, the water adsorption levels of ABZ-FMA, ABZ-DTA, and ABZ-HCl were 6.72%, 70.15%, and 37.65%, respectively. The water adsorption of ABZ-DTA was significant. ABZ had the lowest water adsorption, with a water adsorption rate of only 3.86% at 90% RH. Over the range of 0–90% RH, the weight of ABZ and ABZ-FMA slowly increased, while ABZ-DTA quickly adsorbed water when the RH reached 60%, and the weight began to increase sharply, increasing by 47.59%. ABZ-HCl quickly adsorbed moisture within the initial 0–20% RH range, followed by a slow increase in weight. During the desorption process, the adsorption and desorption curves of ABZ-DTA and ABZ-HCl were inconsistent, resulting in hysteresis. The moisture adsorption process is irreversible [31]. The primary reasons for hysteresis are explained by two factors: the chemical composition and the microstructure of the material. In terms of chemical structure, due to the presence of hydrophilic groups such as hydroxyl groups within the molecules, strong intermolecular forces between water molecules and hydroxyl groups limit the desorption of water molecules during the process. In terms of microstructure, scanning electron microscopy observation uncovered that ABZ-DTA has a porous structure, forming an “ink bottle” effect during the drying process [32], meaning that the water entering the “ink bottle” is difficult to desorb out through the bottleneck (micropores); as a result of that, the desorption curve is located above the adsorption curve on the hygroscopic isotherm line. 

The thermodynamic characteristics of salts can be defined using TGA and DSC. TGA can be employed to assess the water loss rate and degradation temperature of compounds, while DSC can examine the melting point of compounds [33]. Figure 3C illustrates the TGA curves of ABZ, ABZ-FMA, ABZ-DTA, and ABZ-HCl. The weight loss of the four substances took place at approximately 192.3 °C, 135.3 °C, 179.2 °C, and 163.4 °C, respectively. Additionally, ABZ and the three salts exhibited no weight loss before 100 °C, suggesting that none of the four substances had adsorbed any water. As can be seen in the DSC curves in Figure 3D, the ABC salts exhibited sharp endothermic peaks that differed from the starting materials. When the onset of the endothermic peak was taken as the melting point, it could be observed from the DSC curve in Figure 3D that the melting points of salts were elevated compared to the raw materials. ABZ, ABZ-FMA, ABZ-DTA, and ABZ-HCl exhibited characteristic endothermic peaks at approximately 190.5 °C, 171.1 °C, 174.2 °C, and 162.1 °C, suggesting that ABZ, ABZ-FMA, ABZ-DTA, and ABZ-HCl underwent degradation while melting. The sharp endothermic peaks differed from the starting materials, indicating the formation of a new crystalline phase, and no phase transition took place before melting. In addition, compared to ABZ (melting enthalpy of 181.4 J/g), ABZ-FMA, ABZ-DTA, and ABZ-HCl exhibited decreased enthalpies by 27.4% (131.7 J/g), 9.4% (164.4 J/g), and 0.8% (179.9 J/g), respectively. The reduction in the melting point and melting enthalpy of the salts may be caused by the combination of the acid and the drug disturbing the intramolecular forces within the drug itself, lowering the melting point and lattice energy. Crystals with weak attraction for holding molecules together exhibit low melting points, while crystals with strong crystal lattices have high melting points. Ultimately, the weak intramolecular forces facilitated the dissociation of drug molecules when contacting the dissolution medium, triggering improved salt solubility, which is aligned with the subsequent results of the intrinsic dissolution rate [34,35]. 

**Figure 4:**
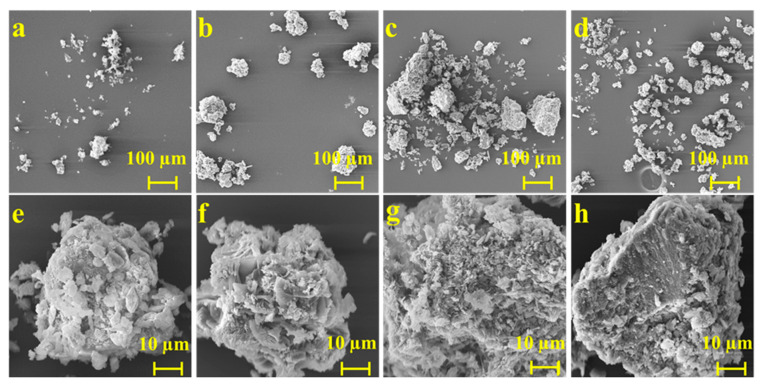
SEM micrographs of ABZ (**a**,**e**), ABZ-FMA (**b**,**f**), ABZ-DTA (**c**,**g**), and ABZ-HCl (**d**,**h**).

SEM can observe the composition and crystal structure of drug salts, obtaining information about the microscopic morphology of substances. Figure 4 illustrates SEM images of ABZ, as well as the three salts, and shows that the morphology and microstructure of each solid particle are highly variable. ABZ aggregates into sheet-like crystals, displaying a compact spherical structure and adhering in a leaf-like formation. Meanwhile, ABZ-FMA exhibits spherical aggregates derived from irregular particles of different sizes and shapes, and layered structures, as well as some pore-like structures, were observed in ABZ-FMA. ABZ-DTA exhibits an irregular crystal formation with a porous morphology and a loose surface, containing many finer pores. ABZ-HCl exhibits a dense and layered structure with irregular small-particle solids attached to the surface.

### 2.2. Solubility

**Figure 5:**
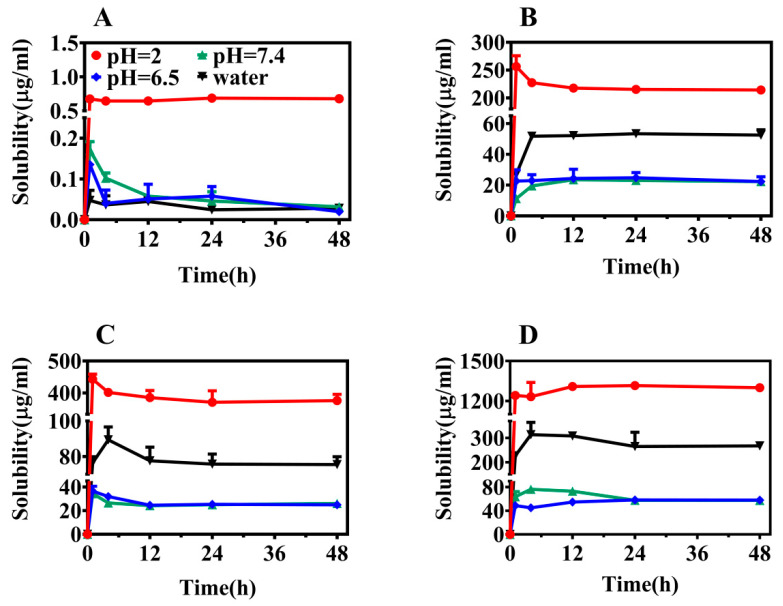
Equilibrium solubility of ABZ (**A**), ABZ-FMA (**B**), ABZ-DTA (**C**), and ABZ-HCl (**D**) under different pH conditions.

The ultimate goal of modification of the crystal structure of a given raw material drug is to enhance its fundamental physicochemical properties, including solubility and the dissolution rate. These factors directly impact the bioavailability of the drug [36]. The solubility of the new solid was evaluated at 37 °C, and Figure 5A–D present dynamic dissolution diagrams of ABZ, ABZ-FMA, ABZ-DTA, and ABZ-HCl in various pH buffers and distilled water solutions.

From the dissolution spectrum, it is clear that all four solid forms reached equilibrium after 24 h, and the solubility of the three salts at different pH conditions was significantly elevated compared to ABZ. In a pH 2.0 buffer, the solubilities of ABZ-FMA, ABZ-DTA, and ABZ-HCl were approximately 330-fold, 585-fold, and 2100-fold higher than that of ABZ, respectively. In a pH 6.5 buffer, the solubilities of ABZ-FMA, ABZ-DTA, and ABZ-HCl were around 480-fold, 500-fold, and 1160-fold higher than ABZ, respectively. The solubilities of ABZ-FMA, ABZ-DTA, and ABZ-HCl in a pH 7.4 buffer were approximately 575-fold, 600-fold, and 1425-fold higher than ABZ, respectively. In distilled water, the solubilities of ABZ-FMA, ABZ-DTA, and ABZ-HCl were over 1000 times higher than that of ABZ. The salt solubility in all four media was significantly higher than ABZ, suggesting that salt formation can improve the solubility of ABZ (especially in distilled water media), with ABZ-HCl being the most advantageous. At the same time, when the solvent alterations moved from an acidic environment to an alkaline environment, the solubility of all four forms was decreased. This phenomenon is attributed to ABZ being a base with a pKa equal to 9.51. As the pH value decreased, the carboxylic acid groups deprotonated, increasing the solubility of the molecule.

### 2.3. Intrinsic Dissolution Rate

**Figure 6:**
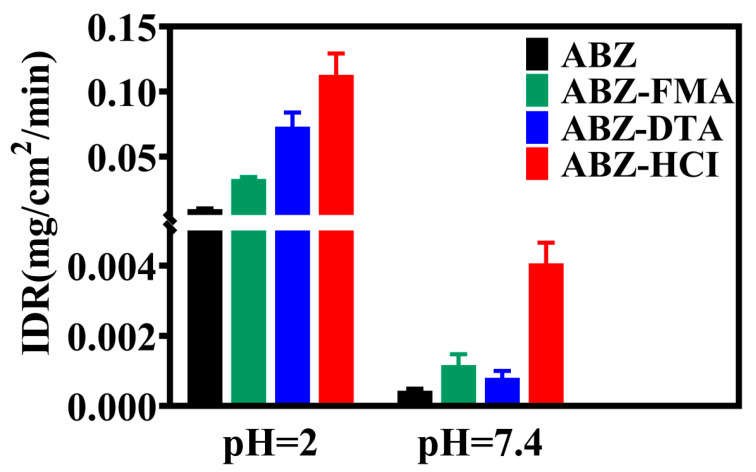
Intrinsic dissolution rates of ABZ, ABZ-FMA, ABZ-DTA, and ABZ-HCl at pH 2 and pH 7.4.

The intrinsic dissolution rate (IDR) represents the inherent dissolution rate of a drug, indicating the mass of a certain amount of drug dissolved per unit area and time in a specific medium [37]. IDR is not a dissolution equilibrium but is closely linked to the dissolution rate. Compared to solubility, it more appropriately reflects the dissolution performance of drugs and has improved correlation with the in vivo pharmacokinetics [38]. The IDR findings of the drug salt are highlighted in Figure 6, showing that the IDRs of ABZ, ABZ-FMA, ABZ-DTA, and ABZ-HCl in pH 2 buffer were 0.0096, 0.0328, 0.0730, and 0.1128 mg·cm^−2^·min^−1^, respectively, which were approximately 3.4-, 7.6-, and 11.8-fold higher than ABZ. The IDRs of ABZ, ABZ-FMA, ABZ-DTA, and ABZ-HCl in pH 7.4 buffer were 0.0004, 0.0009, 0.0008, and 0.0041 mg·cm^−2^·min^−1^, respectively, approximately 2.3-, 2.0-, and 10.3-fold higher than ABZ, respectively.

From the above findings, all three ABZ salts could increase the IDR of ABZ and exhibit an optimal dissolution effect in acidic media (pH 2). The improved dissolution effect of salt can be attributed to the following three reasons: (1) Alterations in lattice energy. The greater the lattice energy, the higher the stability of the crystal, and the stronger the interaction between its molecules, corresponding to lower solubility. It was identified from the DSC results that the melting point and melting enthalpy of the salts were reduced compared to ABZ, meaning that the lattice energy was diminished, resulting in facilitated dissociation of drug molecules when contacting the dissolution medium. (2) Changes in micro-morphology. In general, looser structures (such as a porous morphology) can provide increased surface area, a greater chance of contact with the dissolution medium, a better dissolution effect, and a higher dissolution rate than compact structures. In the SEM results, ABZ-DTA exhibits a porous structure (as shown in the scanning electron microscope image in Figure 4), possibly contributing to its improved salt dissolution rate [28]. (3) The pH value of the local microenvironment. The pH value of the local microenvironment has an important impact on the dissolution of drugs. The pKa of fumaric acid is 3.02, the pKa of D-tartaric acid is 3.00, and the pKa of hydrochloric acid is −7.00. Using appropriate counter ions to enable the optimal pH for salt can enhance the dissolution rate of drugs, while hydrochloric acid provides an optimally acidic pH [39,40].

### 2.4. Solid-State Stability

#### 2.4.1. Influencing Factor Testing

**Figure 7:**
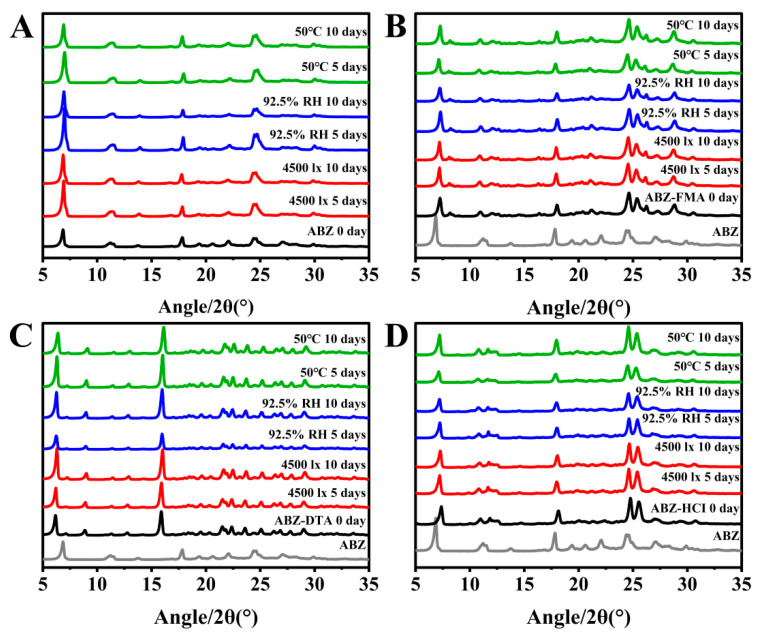
Experimental PXRD results at 0, 5, and 10 days post-conditioning at 4500 lux, 92.5% RH, and 50 °C in samples of ABZ (**A**), ABZ-FMA (**B**), ABZ-DTA (**C**), and ABZ-HCl (**D**).

**Figure 8:**
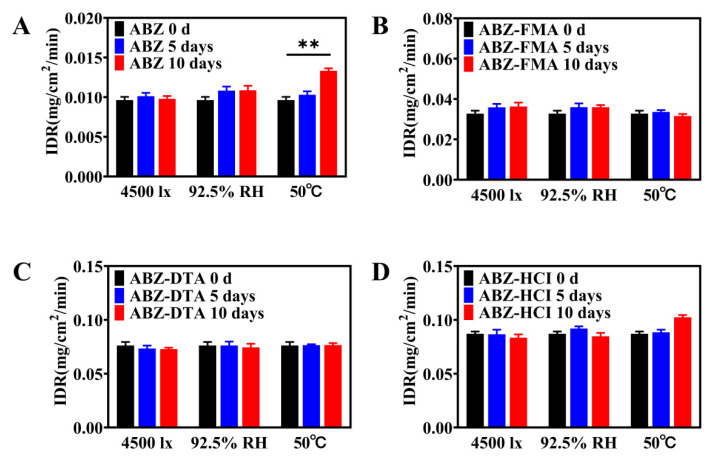
Intrinsic dissolution rate results at 0, 5, and 10 days post-conditioning at 4500 lux, 92.5% RH, and 50 °C in samples of ABZ (**A**), ABZ-FMA (**B**), ABZ-DTA (**C**), and ABZ-HCl (**D**) (**: *p* < 0.01).

To examine the solid-state stability of salts, the morphology and dissolution behavior of drug salt crystals were observed following the exposure of ABZ, ABZ-FMA, ABZ-DTA, and ABZ-HCl to strong light (4500 ± 500 lux), high humidity (92.5 ± 5% RH), and elevated temperature (50 ± 2 °C). Figure 7A–D illustrate the PXRD spectra. The crystal transformation phenomena in ABZ-FMA, ABZ-DTA, and ABZ-HCl were not observed. At the same time, the IDRs of ABZ and different salts under different conditions after 0, 5, and 10 days were compared, as highlighted in Figure 8A–D. After being placed at 50 °C, the IDR of ABZ increased, indicating an unstable dissolution behavior. However, there was no statistically significant difference in IDR on the 5th and 10th days when observed in ABZ-FMA, ABZ-DTA, and ABZ-HCl under strong light exposure, high humidity, and high-temperature conditions compared to day 0. This indicates that strong light, high humidity, and elevated temperature conditions do not impact the dissolution of the salt, and that ABZ salt formulations can enhance the thermal stability of ABZ. 

#### 2.4.2. Accelerated Stability

An accelerated stability study was conducted on ABZ, ABZ-FMA, ABZ-DTA, and ABZ-HCl samples at 40 ± 2 °C/75 ± 5% RH. Solid samples were obtained at 0, 3, and 6 months under these conditions and examined by PXRD.

**Figure 9:**
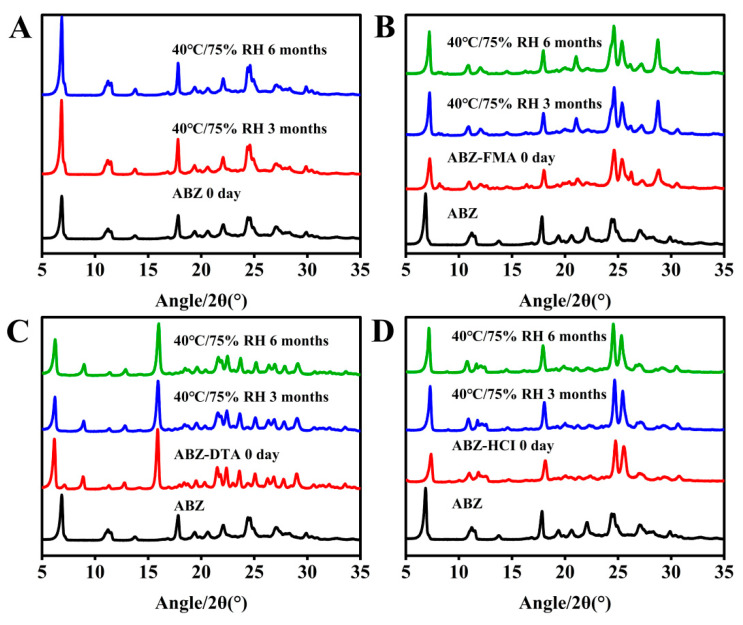
Experimental PXRD results at 0, 3, and 6 months post-conditioning at 40 °C/75% RH in samples of ABZ (**A**), ABZ-FMA (**B**), ABZ-DTA (**C**), and ABZ-HCl (**D**).

Figure 9A–D illustrate the PXRD spectra of the four solid forms. According to Figure 8B–D, the PXRD spectra of ABZ-FMA, ABZ-DTA, and ABZ-HCl in the accelerated stability study conditions were unchanged, indicating appropriate physical stability of ABZ-FMA, ABZ-DTA, and ABZ-HCl.

### 2.5. Pharmacokinetic Comparison in Wistar Rats

To examine the oral absorption of ABZ salts in a half-male and half-female group of Wistar rats, ABZ, ABZ-FMA, ABZ-DTA, and ABZ-HCl were orally administered at a dose of 25 mg/kg. The bioavailability of the three salts and the prototype drug were contrasted to identify whether ABZ salt had solubilization effects in vivo.

**Figure 10:**
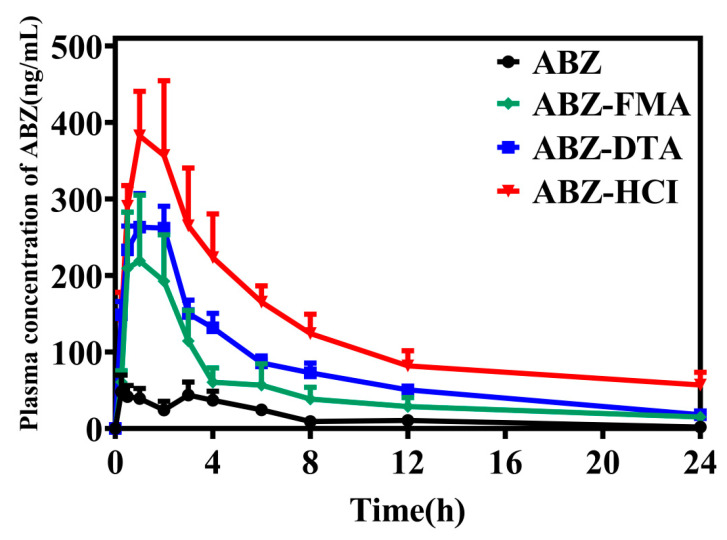
Drug release investigation of the ABZ formulation in vivo.

Following oral administration of ABZ and drug salts, the salt enhanced the absorption of ABZ (the concentration of ABZ was nearly undetectable in the raw material group after 24 h). The oral absorption effects of the four solid forms were ABZ-HCl > ABZ-DTA > ABZ-FMA > ABZ, corresponding to the in vitro dissolution results. As illustrated in Figure 10 and Table 1, the C_max_ of ABZ, ABZ-FMA, ABZ-DTA, and ABZ-HCl were 69.61 ± 51.71 ng/mL, 260.58 ± 179.73 ng/mL, 322.68 ± 68.67 ng/mL, and 478.96 ± 138.62 ng/mL, respectively. Compared to ABZ, ABZ-FMA, ABZ-DTA, and ABZ-HCl exhibited increased C_max_ values by approximately 3.8, 4.6, and 6.9 times, respectively. Correspondingly, the bioavailability of ABZ was significantly enhanced after salt preparation, and the area under the plasma concentration–time curve was significantly elevated. The AUC_(0–24)_ of ABZ-FMA, ABZ-DTA, and ABZ-HCl were 1205.56 ± 855.61 ng/mL/hour, 1835.37 ± 379.84 ng/mL/hour, and 3089.28 ± 1228.16 ng/mL/hour, respectively, which were about 3.4, 5.2, and 8.8 times that of ABZ (352.44 ± 146.70 ng/mL/hour). Table 1 outlines all pharmacokinetic parameters. These findings suggest that the solubility of ABZ was improved by preparing it as a salt, significantly increasing the oral bioavailability and absorption rate. Additionally, the T_max_ of the three salts was observed earlier than ABZ, in the range of 0.87 to 1.33 h. The difference in T_max_ may be caused by the increased solubility of ABZ compared to API, resulting in an increase in peak plasma concentration and bioavailability.

## 3. Materials and Methods

### 3.1. Materials and Reagents

Albendazole (purity > 98.0%) was obtained from Beijing Ouhe Technology Co., Ltd. (Beijing, China). Fumaric acid (purity > 98.0%), D-tartaric acid (purity > 98.0%), and hydrochloric acid (purity > 98.0%) were purchased from Shanghai McLean Biotechnology Co., Ltd. (Shanghai, China). Methanol and chloroform (analytical reagent grade) were acquired from the Tianjin Damao chemical reagent factory (Tianjin, China). These were employed in the crystallization of salts. 

### 3.2. Preparation of Albendazole Salts

Equimolar amounts of albendazole and fumaric acid, D-tartaric acid, and hydrochloric acid were dissolved in a chloroform/methanol (7:3) solution, and stirred thoroughly for 2 h at 25 °C. Through evaporation of the solvent via rotary evaporation (at 50 °C and 80 rpm), the solvent was removed. The solid powder was vacuum-dried for 24 h, and following grinding, ABZ-FMA, ABZ-DTA, and ABZ-HCl salt powders were generated.

### 3.3. Characterization of Albendazole Salts

#### 3.3.1. Fourier Transform Infrared Spectroscopy (FT-IR)

FT-IR analysis was conducted using a Fourier transform infrared spectrophotometer (Nicolet 6700, Thermo Electron Corporation, Waltham, MA, USA). Scans were performed within the range of 4000 to 400 cm^−1^ at room temperature, using a spectral acquisition resolution of 4 cm^−1^.

#### 3.3.2. Nuclear Magnetic Resonance (^1^H-NMR)

The ^1^H NMR spectrum was acquired using an AVANCE NEO 600 spectrometer (Bruker BioSpin GmbH, Rheinstetten, Germany) using a working frequency of 600 MHz. The solid sample was completely dissolved in deuterated DMSO (DMSO-d6), and the prepared samples were analyzed at 25 °C.

#### 3.3.3. Powder X-ray Diffraction (PXRD)

An appropriate amount of sample was weighed and analyzed on a*–D-max2500PC X-ray Diffractometer (Nihonomi, Kobe, Japan) using Cu-Kα target determination (λ = 1.54 Å), a scanning range of 2θ = 5 to 35°, scanning steps of 0.01°, a scanning speed of 1°/min, a voltage of 40 kV, and a current of 200 mA.

#### 3.3.4. Dynamic Vapor Sorption (DVS)

The moisture sorption characteristics of all specimens were assessed at a temperature of 25 °C using an automated dynamic vapor sorption analyzer (Surface Measurement Systems, Wembley, UK). Prior to the sorption examination, the samples were equilibrated at a humidity level of 0% relative humidity (RH) to remove any adsorbed moisture. The specimens underwent a gradual variation in relative humidity, transitioning from 0 to 90% RH at intervals of 10% RH. Humidity was adjusted by increasing it by +10% RH after 6 h or upon achieving the equilibrium condition. The equilibrium condition was met when there was a <0.002% change in mass per minute within a span of 10 min. Upon completion of the sorption sequence, all samples were subjected to drying at 0% RH, which was stopped when the Δm/Δt was less than 0.002%/min within 10 min.

#### 3.3.5. Thermogravimetric Analysis (TGA)

Approximately 5–10 mg of the sample was weighed on an aluminum plate and analyzed using a NETZSCH synchronous thermal analyzer (STA449F3-DSC200F3, Netzsch, Waldkraiburg, Germany). The temperature range was between 30 and 300 °C, with a heating rate of 10 °C/min, and we used a nitrogen flow rate of 50 mL/min.

#### 3.3.6. Differential Scanning Calorimetry (DSC)

The melting point (*T_m_*) of salts was identified using an identical thermal analyzer as described above. Briefly, approximately 5–10 mg of the sample was weighed using a balance, placed on an aluminum plate, and heated from 30 °C to 300 °C at a rate of 10 °C/min.

#### 3.3.7. Scanning Electron Microscopy (SEM)

The surface appearance and configuration of all specimens were examined with SEM (JSM-6610LV, JEOL LTD, Japan Electronics, Tokyo, Japan). The specimens were affixed to a copper platform and subjected to observation after a 300 s gold-plating process carried out with a sputtering coating apparatus operating in a vacuum environment. The operational voltage was set at 15 kV.

### 3.4. Solubility

To relate the solubility of ABZ and ABZ salts, 30 mg of solid sample was added to a test tube containing 10 mL of HCl buffer (pH = 2.0), phosphate buffer (pH = 6.8), phosphate buffer (pH = 7.4), or distilled water. The solution was vibrated at 100 rpm at a controlled temperature (37 °C ± 1 °C). Samples were obtained at time points corresponding to 0.5 h, 1 h, 4 h, 12 h, 24 h, and 48 h, and the supernatant was obtained via centrifugation. The drug concentration was determined through High-Performance Liquid Chromatography (HPLC) after dilution with methanol. HPLC analysis was conducted using an Agilent 1260 Series high-performance liquid chromatograph (Agilent Technologies, Santa Clara, CA, USA). The chromatographic conditions were as follows: the mobile phase consisted of methanol/water (80/20 *v*/*v*), an octadecylsilane chromatographic column (Diamonsil C18, 250 mm × 4.6 mm, 5 µm) was used as a stationary phase, and a UV wavelength of 295 nm was used, along with a flow rate 1 mL/min, a column temperature of 30 °C, and an injection volume of 20 µL.

### 3.5. Intrinsic Dissolution Rate (IDR)

The powdered sample (140 mg) was measured and compacted to a tablet hardness of 5 Kg by a tester, employing a small manual tablet press. The compressed tablets were sealed in paraffin, with only one side exposed to dissolution solution (20 mL, 37 °C), which is a mixture of 0.01 M HCl (pH = 2.0) and PBS (pH = 7.4). Then, 0.3 mL of the solution was withdrawn, at 1 min intervals, and subsequently subjected to centrifugation (15,000 rpm, 3 min). Afterward, it was subjected to double dilution with methanol and analyzed for the presence of the drug using HPLC.

### 3.6. Solid-State Stability

#### 3.6.1. Influencing Factor Testing

Influencing factor testing included a strong light irradiation test, a high-humidity test, and an elevated temperature test. The strong light irradiation test involved placement of the sample opening under a light with an intensity of 4500 ± 500 lux. The high-humidity test involved placement of the sample in a constant-humidity container at 25 °C and 92.5% RH (saturated potassium nitrate solution). The high-temperature test involved placing the sample in a DHG-9070A constant-temperature oven (Shanghai Yiheng Technology Instrument Co., Ltd., Shanghai, China), set at 50 °C. Samples were taken on the 0th, 5th, and 10th days, and the crystal structure and dissolution of the samples were assessed for significant differences based on PXRD and IDR, as outlined above.

#### 3.6.2. Accelerated Testing

Accelerated testing was utilized to contrast the stability of ABZ and its salts in the solid state. The solid variations were placed in an airtight container at 40 ± 2 °C with a humidity level of 75 ± 5% RH, and specimens were harvested at the intervals of 0, 3, and 6 months. PXRD analysis was used to ascertain whether there were any substantial alterations in dissolution properties and the crystal structure of the sample.

### 3.7. Pharmacokinetic Comparison in Wistar Rats

In vivo pharmacokinetic examination of albendazole and albendazole salts was performed in a half-male and half-female group of Wistar rats (*n* = 6), with an average body weight of 200 ± 20 g. The rats were fasted for one night before administration of albendazole at a dose of 25 mg/kg by gavage. Blood sampling was conducted by placing a jugular vein catheter surgically the day before administration. At 0.25 h, 0.5 h, 1 h, 2 h, 3 h, 4 h, 6 h, 8 h, 12 h, and 24 h after administration, samples of 300 to 500 μL of blood were collected into pre-prepared anticoagulant-coated tubes. The tubes were centrifuged at 13,000× *g* for 10 min, and the upper plasma was collected and stored in an ultra-low-temperature freezer at −80 °C. Samples were processed by obtaining 100 μL of plasma; then, 400 μL of methanol was added, the samples were vortexed for 1 min, and they were centrifuged at 13,000× *g* for 5 min. Finally, 300 μL of the supernatant was obtained. A mixture of 6 μL of 10 μG/mL toluidazole internal standard solution was added to the supernatant. After 1 min of vortexing, the samples were centrifuged at 13,000× *g* for 5 min. The supernatant was then filtered through a microporous filter membrane before employing UPLC-HR-ESI-MS to assess the drug content.

The plasma concentrations of ABZ were assessed using a Dionex Ultimate 3000 RSLC system (Thermo Fisher, Waltham, MA, USA) equipped with an ACQUITY UPLC BEH C18 Column (130 Å, 150 mm × 2.1 mm × 2.6 μm) at a column temperature of 30 ± 1 °C. The mobile phase consisted of eluent A (HPLC-grade acetonitrile supplemented with 0.1% FA) and eluent B (water supplemented with 0.1% FA) and was delivered in a gradient elution mode as follows: 0 to 6 min, 8% to 40% B; 6 to 8 min, 40% to 100% B; 8 to 10 min, 100% B; 10 to 11 min, 100% to 8% B; and 11 to 13 min, 8% B, all at a flow rate of 0.3 mL/min. The MS analysis was conducted using a Q-Orbitrap MS coupled with heated electrospray ionization (HESI, Washington, DC, USA). The MS analysis was performed in full MS-ddMS^2^ mode, with damping gas in the C-trap and nitrogen used to stabilize the spray. The auxiliary gas heater and capillary temperatures were established and maintained at 350 °C and 320 °C, respectively. In the negative mode, a spray voltage of 3.0 kV and an S-lens RF level of 60 V were employed. The full MS-ddMS^2^ scan range was from 150.0000 to 1000.0000 *m*/*z*, and the precise molecular weight [M − H]^+^ was employed for qualitative analysis. The corresponding peak area was utilized for quantitative analysis, and the MS^2^ fragments were employed in further qualitative analysis.

The pharmacokinetic parameters were examined through non-compartment model analysis using DAS 2.0.

## 4. Conclusions

In this study, we prepared crystalline albendazole fumarate, albendazole D-tartrate, and albendazole hydrochloride (ABZ-FMA, ABZ-DTA, and ABZ-HCl) using a solvent evaporation method. We extensively characterized these salts through various techniques, providing detailed data on novel solid forms. The shift in characteristic peaks was identified in FT-IR and ^1^H NMR, suggesting intermolecular interactions between carbamate and benzimidazole groups in the ABZ structure, verifying the formation of salts. PXRD, DVS, and thermal analysis (TGA and DSC) established the crystallization, water adsorption, and thermodynamic properties of ABZ-FMA, ABZ-DTA, and ABZ-HCl. Simultaneously, SEM analysis of the microstructure reinforced the dissolution properties of the drug salt. Following the preparation of ABZ as a salt, its water solubility was improved. The solubility and intrinsic dissolution rate of ABZ-FMA, ABZ-DTA, and ABZ-HCl were elevated compared to ABZ under varied pH conditions, suggesting that all three salts improved the solubility and dissolution rate of ABZ, with ABZ-HCl having the most improved solubility performance. ABZ-FMA, ABZ-DTA, and ABZ-HCl remained stable for 10 days under strong light irradiation (4500 ± 500 lux), high humidity (92.5 ± 5% RH), and elevated temperature conditions (50 ± 2 °C). Additionally, they remained stable for 6 months under accelerated test conditions (40 °C/75 ± 5% RH), suggesting adequate drug salt stability. With the improvement in solubility, ABZ salt exhibits improved oral bioavailability and pharmacokinetic properties, indicating its potential application in the oral administration of ABZ.

The salts identified in this study not only expand the diversity of solid forms of ABZ, providing useful information for improving the solubility and oral bioavailability of ABZ, but also provide options for the next stage of drug formulation development. Moreover, these findings offer the possibility for further identification of novel solid forms of ABZ capable of improving solubility, stability, and oral bioavailability.

## Figures and Tables

**Table 1:** Pharmacokinetics parameters of ABZ, ABZ-FMA, ABZ-DTA, and ABZ-HCl in vivo.

Parameters	ABZ	ABZ-FMA	ABZ-DTA	ABZ-HCI
AUC_(0–24)_ (ng/mL/h)	352.44 ± 146.70	1205.56 ± 855.61	1835.37 ± 379.84	3089.28 ± 1228.16
AUC_(0–∞)_ (ng/mL/h)	374.31 ± 135.98	1975.42 ± 1856.96	1955.94 ± 431.79	5371.96 ± 4618.85
C_max_ (ng/mL)	69.61 ± 51.71	260.58 ± 179.73	322.68 ± 68.67	478.96 ± 138.62
T_max_ (hours)	2.54 ± 1.57	1.67 ± 1.17	1.25 ± 0.61	1.21 ± 0.68
T_1/2_ (hours)	5.98 ± 3.27	14.07 ± 14.37	6.11 ± 1.21	16.85 ± 23.17
CL_Z/F_ (L/h/kg)	73,576.20 ± 23,270.84	25,882.64 ± 19,061.91	13,340.60 ± 3054.71	7454.90 ± 4568.17
MRT_(0–24)_ (hours)	7.07 ± 2.27	6.41 ± 0.66	6.67 ± 0.61	7.66 ± 2.08
MRT_(0–∞)_ (hours)	9.19 ± 5.10	17.53 ± 15.90	8.92 ± 1.51	24.65 ± 33.76

## Data Availability

The data that support the findings of this study are available from the author, Haiying Yan, upon reasonable request.
